# Supplementary material for: An iron (II) dependent oxygenase performs the last missing step of plant lysine catabolism
Source: Nat Commun. 2020 Jun 10;11:2931. doi: 10.1038/s41467-020-16815-3 (PMC7286885; doi:10.1038/s41467-020-16815-3)
Supplement: Supplementary file 1 — Supplementary Information [file 41467_2020_16815_MOESM1_ESM.pdf]

An iron (II) dependent oxygenase performs the last missing step of plant lysine catabolism

Mitchell G. Thompson,[1,2,3*] Jacquelyn M. Blake-Hedges,[1,2,4*] Jose Henrique Pereira,[1,5*] John A. Hangasky,[4] Michael S. Belcher,[1,2,3] William M. Moore,[1,2,3] Jesus F. Barajas,[1,2,6] Pablo Cruz-Morales,[1,2,] Lorenzo J. Washington,[1,2,3] Robert W. Haushalter,[1,2] Christopher B. Eiben,[1,2,711], Yuzhong Liu,[1,2] Will Skyrud,[4] Veronica T. Benites,[1,2] Tyler P. Barnum,[3] Edward E. K. Baidoo,[1,2] Henrik V. Scheller,[1,2,3] Michael A. Marletta,[4,87] Patrick M. Shih,[1,2,8,9,10,11] Paul D. Adams,[1,5,711] Jay D. Keasling[1,2,711,12,13,14,#]

[1]Joint BioEnergy Institute, Emeryville, CA, USA
[2]Biological Systems & Engineering Division, Lawrence Berkeley National Laboratory, Berkeley, CA, USA
[3]Department of Plant and Microbial Biology, University of California-Berkeley, Berkeley, CA, USA
[4]Department of Chemistry, University of California-Berkeley, Berkeley, CA, USA
[5]Molecular Biophysics and Integrated Bioimaging, Lawrence Berkeley National Laboratory, Berkeley, CA, USA
[6]Department of Energy Agile BioFoundry, Emeryville, CA, USA
[7]Department of Bioengineering, University of California-Berkeley, Berkeley, CA 94720, USA
[87]Department of Molecular and Cellular Biology, University of California-Berkeley, Berkeley, CA, USA
[98]Department of Plant Biology, University of California-Davis, Davis, CA, USA
[109]Genome Center, University of California-Davis, Davis, CA, USA
[1110]Environmental Genomics and Systems Biology Division, Lawrence Berkeley National Laboratory, Berkeley, CA, USA
[11]Department of Bioengineering, University of California-Berkeley, Berkeley, CA 94720, USA
[12]Department of Chemical and Biomolecular Engineering, University of California-Berkeley, Berkeley, CA, USA
[13]The Novo Nordisk Foundation Center for Biosustainability, Technical University of Denmark, Denmark
[14]Center for Synthetic Biochemistry, Shenzhen Institutes for Advanced Technologies, Shenzhen, China
*Authors contributed equally
# Corresponding author jdkeasling@lbl.gov

Supplementary Information

Supplementary Figures

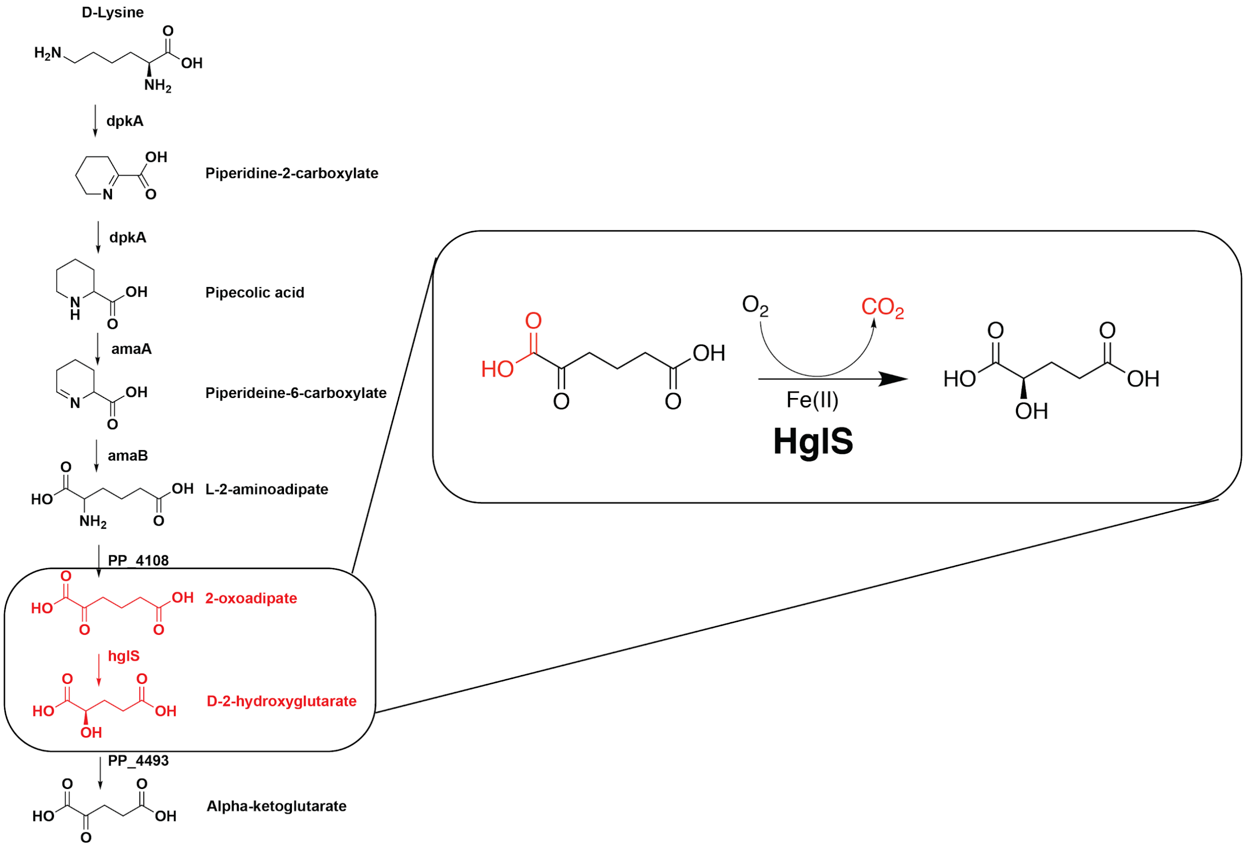

Supplementary Figure 1: D-lysine catabolic pathway of *Pseudomonas putida*. D-lysine is catabolized independently of L-lysine in *Pseudomonas putida*. PP_4108 transaminates 2AA to 2OA. HglS then catalyzes a previously undescribed 2OA to D-2HG decarboxylation (see inset).[1] D-2HG is then oxidized to alpha-ketoglutarate, connecting lysine metabolism to central metabolism (the TCA cycle).

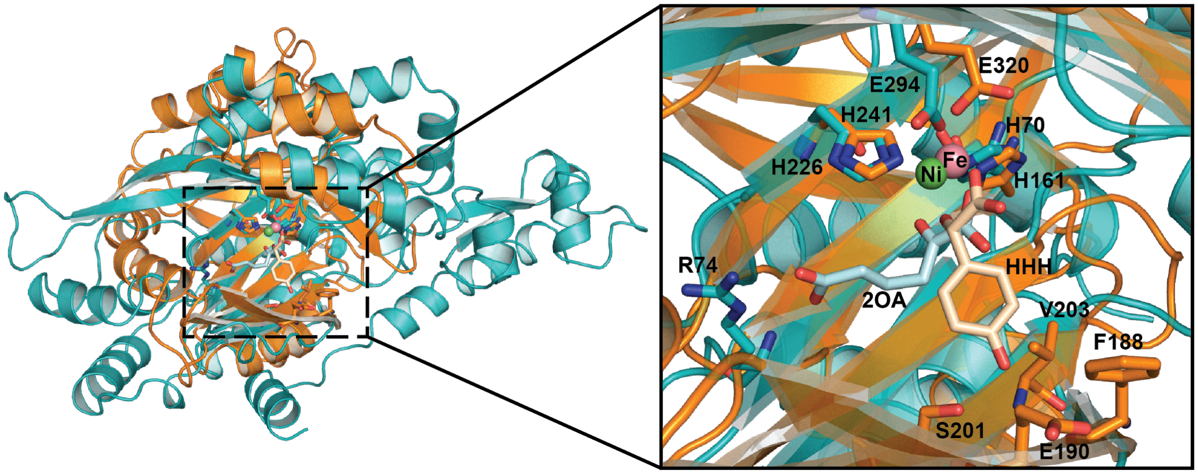

Supplementary Figure 2: Overlay of HglS and HMS. Overlay of the active sites of HglS (teal) and HMS (orange) showing the conservation of the central β-sheet domain and active site architecture. The inset shows a magnified image of the active site in which metal-binding residues, substrates, and residues that interact with substrates in either enzyme are shown as sticks. The metal cofactors for HglS (green) and HMS (pink) are labeled with the appropriate chemical symbol. The substrates are labeled as 2OA (2-oxoadipate) and HHH ((2S)-hydroxy(4-hydroxyphenyl)ethanoic acid) and shown in light blue and tan, respectively.

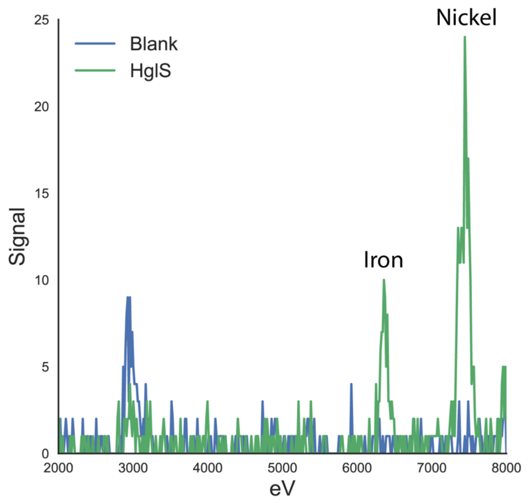

Supplementary Figure 3: Kα emission spectrum of HglS. K-alpha emission spectrum resulting from a fluorescent scan of an HglS crystal (green line), or buffer blank (blue line). The emitted wavelengths were characteristic of Ni (near 7475 eV), as well as Fe (near 6400 eV).

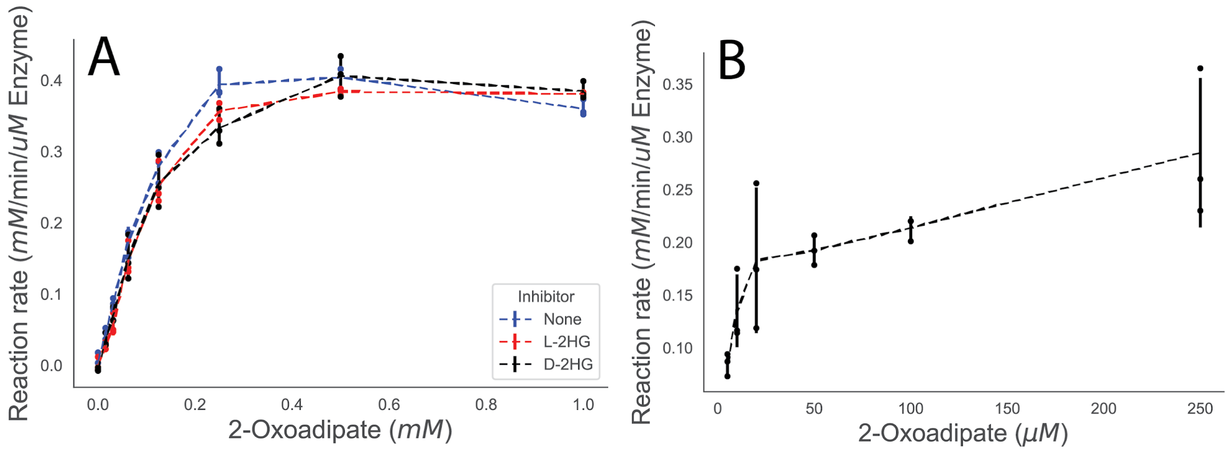

Supplementary Figure 4: Feedback inhibition and O_2 kinetics of HglS. A: Reaction rates of HglS on 2OA in the presence of either L-2HG (red) or D-2HG (back) compared to a control with no added product (blue) as measured by enzyme coupled decarboxylation assay, n=3. Error bars represent 95% confidence intervals, dots represent individual measurements. **B**: Reaction rates of HglS measured by oxygen consumption, n=3. Error bars represent 95% confidence intervals, dots represent individual measurements. The K_m was determined to be 0.01 mM with a Vmax of 0.273 mM/min/µM enzyme.

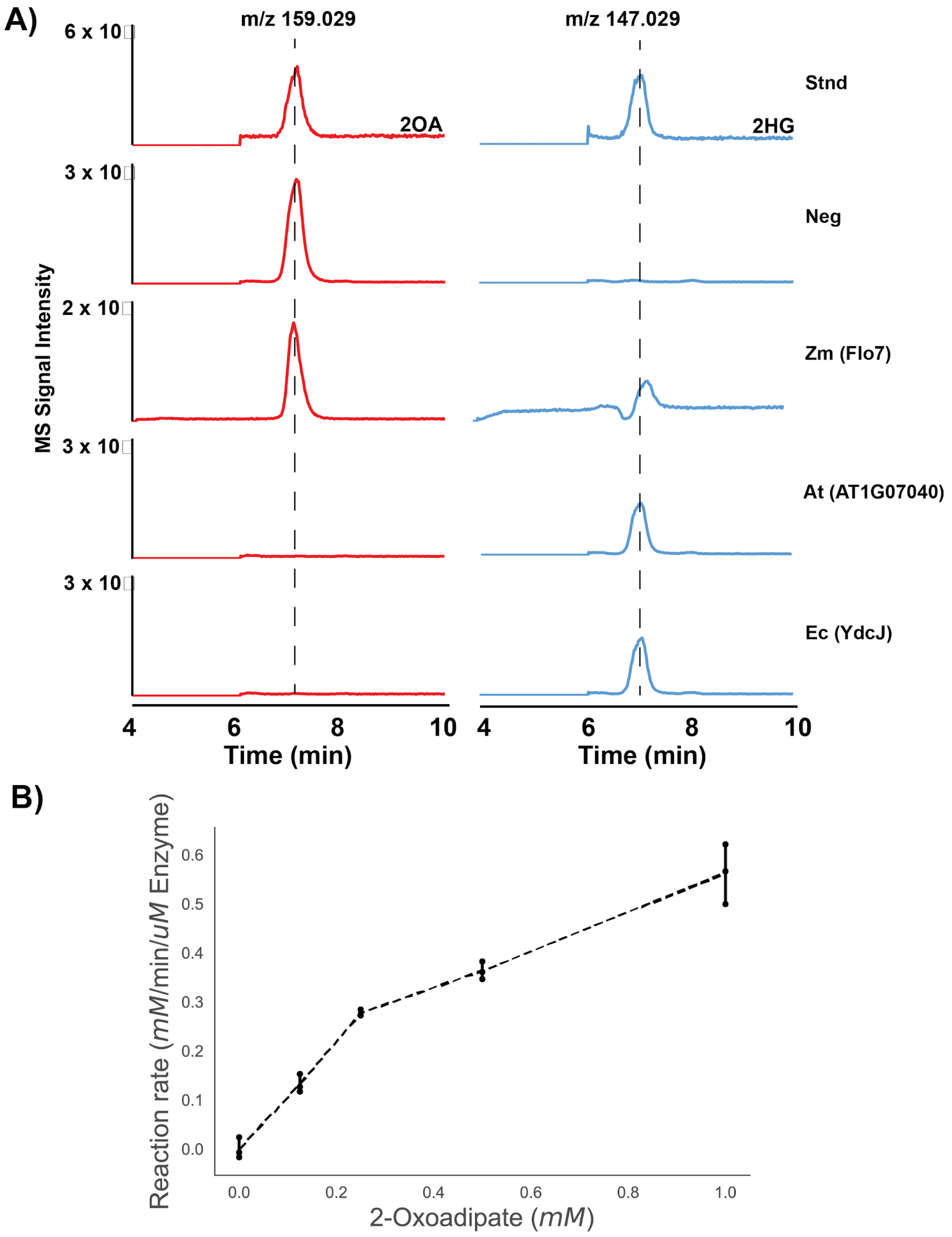

Supplementary Figure 5 - Biochemical analysis of plant HglS homologs: A) Extracted ion chromatograms (EICs) from high resolution LC-TOF analysis of biochemical assays of HglS homologs performed in negative ion mode. EICs pertaining to 2-oxoadipate (2OA, m/z 159.029,

left column) are shown in red, while EICs pertaining to 2-hydroxyglutarate (2HG, m/z 147.029, right column) are shown in blue. Assays were compared to authentic standards (Stnd) and negative control reactions (Neg) containing no enzyme. Data shown pertain to assays performed with the following homologs: *Zea mays*/Flo7 (Zm(Flo7)), *Arabidopsis thaliana*/AT1G07040 (At(AT1g07040)), and *Escherichia coli*/YdcJ (Ec(YdcJ)). The chromatograms shown are a representative sample from assays performed in triplicate. **B)** Flo7 enzyme turnover rates when provided with varying concentrations of 2OA substrate as measured by an enzyme coupled assay. Error bars represent 95% confidence interval, dots represent individual measurements, n = 3

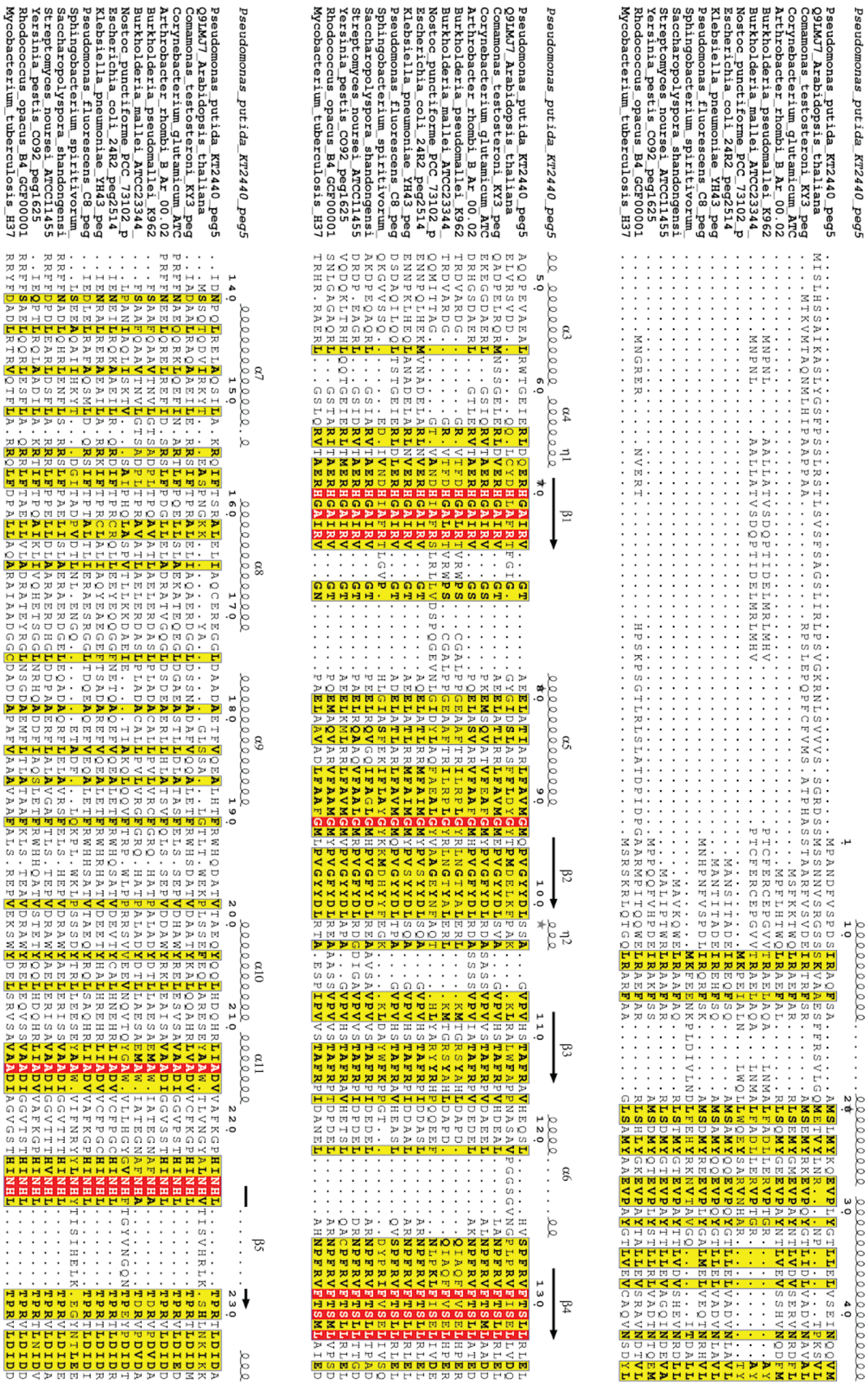

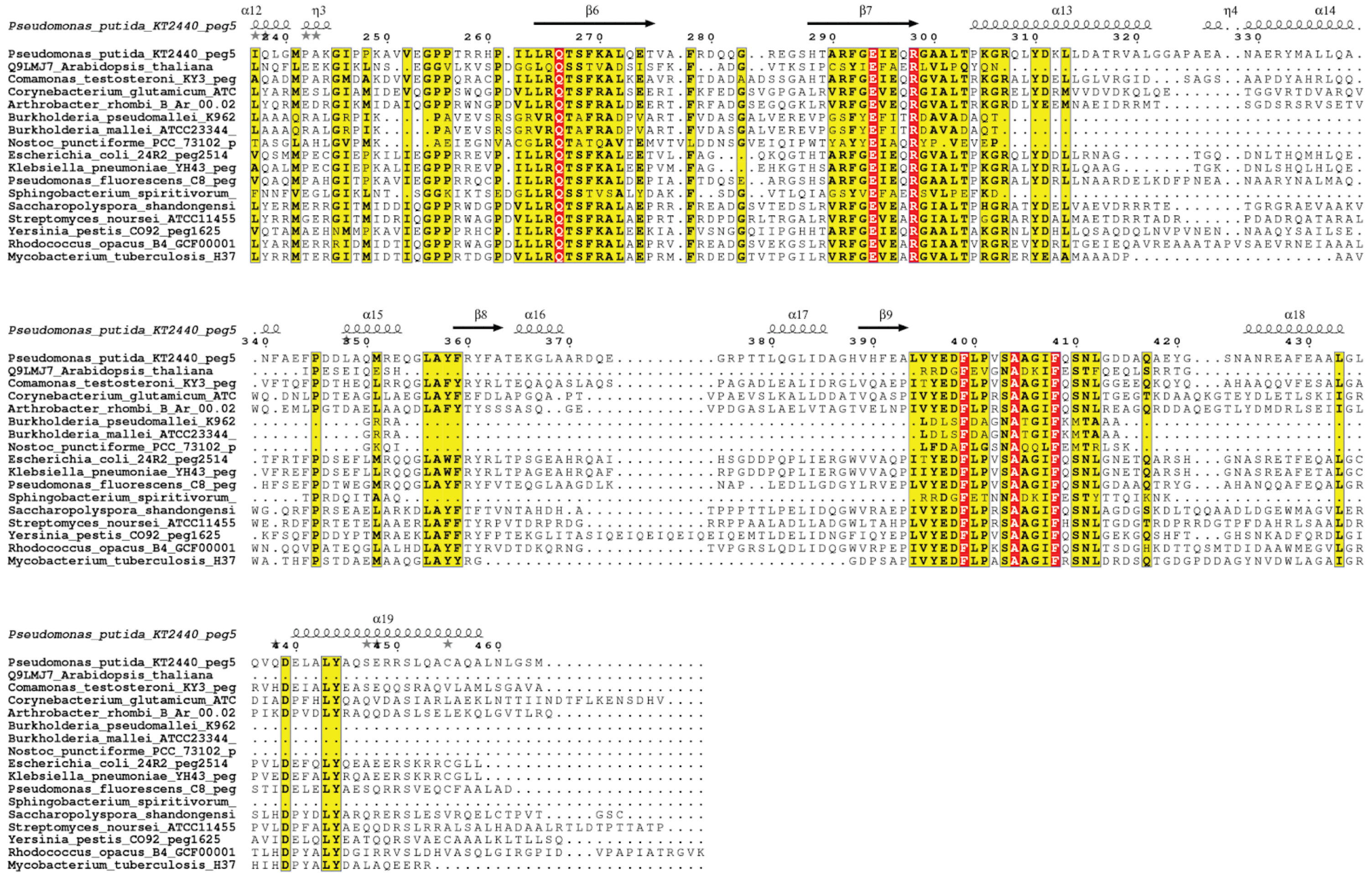
Pseudomonas_putida_KT2440_peg5
Q9LMJ7_Arabidopsis_thaliana
Comamonas_testosteroni_KY3_peg
Corynebacterium_glutamicum_ATC
Arthrobacter_rhombi_B_Ar_00.02
Burkholderia_pseudomallei_K962
Burkholderia_mallei_ATCC23344_
Nostoc_punctiforme_PCC_73102_p
Escherichia_coli_24R2_peg2514
Klebsiella_pneumoniae_YH43_peg
Pseudomonas_fluorescens_C8_peg
Sphingobacterium_spiritivorum_
Saccharopolyspora_shandongensi
Streptomyces_noursei_ATCC11455
Yersinia_pestis_CO92_peg1625
Rhodococcus_opacus_B4_GCF00001
Mycobacterium_tuberculosis_H37
α12 η3 β6 β7 α13 η4 α14
240 250 260 270 280 290 300 310 320 330
α15 β8 α16 α17 β9 α18
340 350 360 370 380 390 400 410 420 430
α19
440 450 460

Supplementary Figure 6 - Alignment of bacterial and plant homologs of *P. putida* HglS (PP_5260). Conserved residues are shown in yellow, while perfectly conserved residues are shown in red. Above alignments is the secondary structure of HglS.

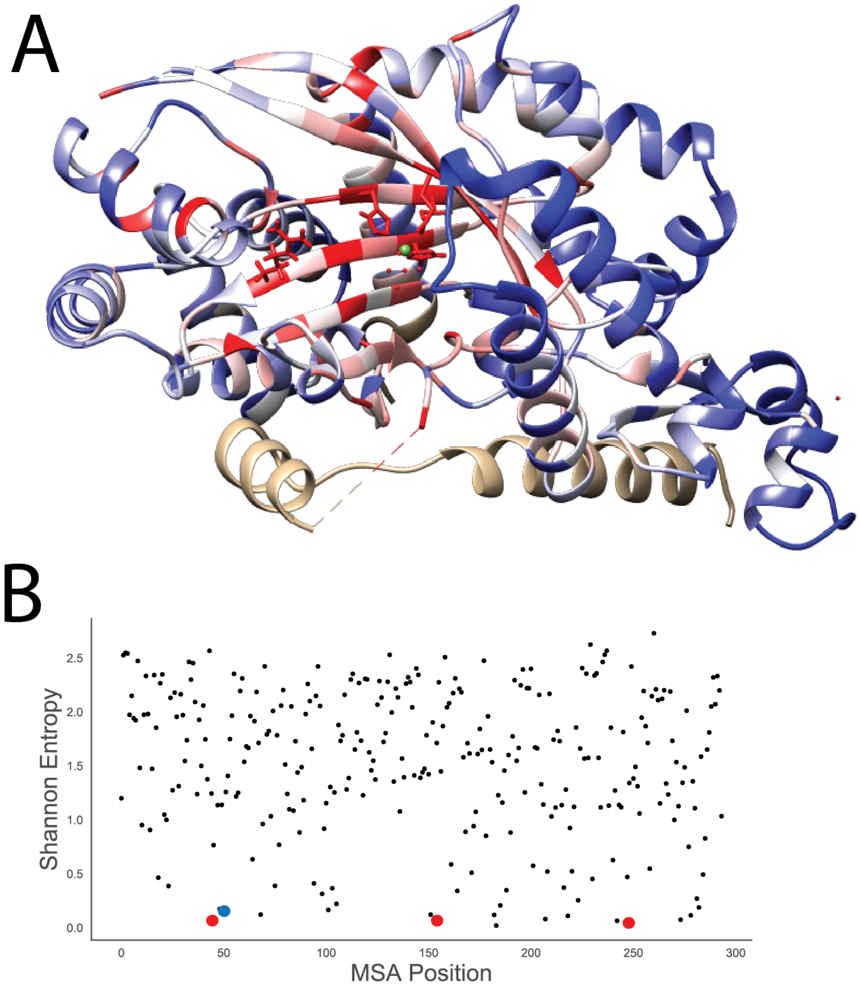

Supplementary Figure 7 - Conservation of critical residues in DUF1338 proteins. A) Structure of HglS with residues colored by conservation, red showing the highest level of conservation with blue showing the least amount of conservation B) Shannon entropy of an alignment of the representative DUF1338 containing proteins in the Pfam database. The Fe (II) coordinating HHE triad residues are highlighted as red dots, while the carboxylate coordinating arginine is highlighted as a blue dot.

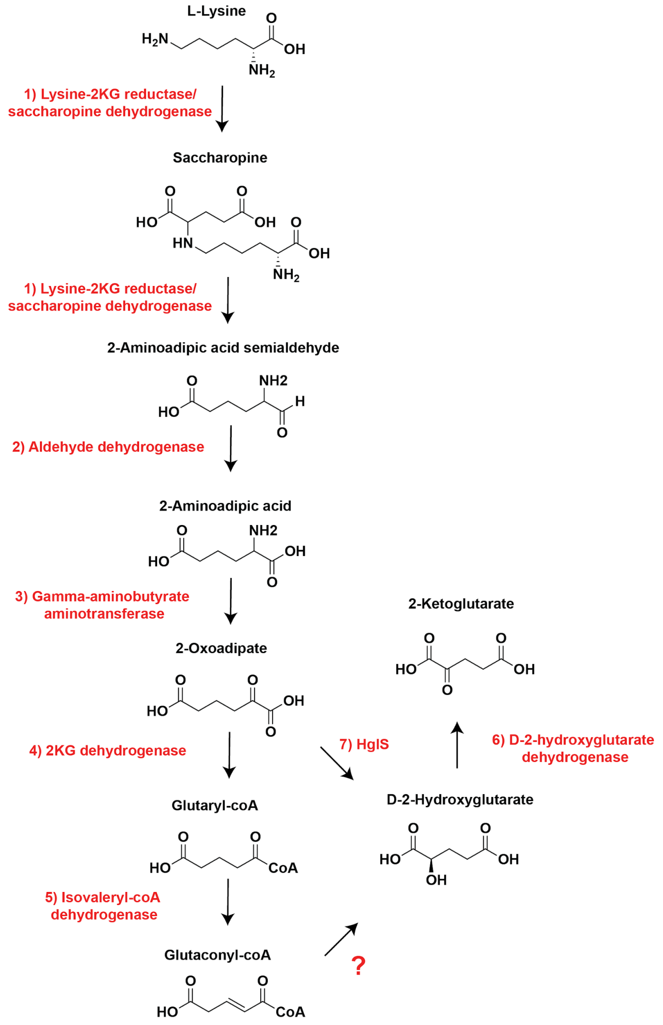

Supplementary Figure 8 - Putative route of lysine catabolism in plants. Enzymes putatively involved in plant lysine catabolism according to [2] 1) Lysine-2KG reductase/saccharopine dehydrogenase 2) Aldehyde dehydrogenase 3) Gamma-aminobutyrate aminotransferase 4) 2KGD 5) Isovaleryl-coA dehydrogenase 6) D-2-hydroxyglutarate dehydrogenase. ? represents a putative enzyme that would convert glutaconyl-coA to D2HG. We propose that HglS (Hydroxyglutarate synthase) homologs (7) catalyze the conversion of 2OA to 2HG in plants, rather than proceeding through the 2KGD complex.

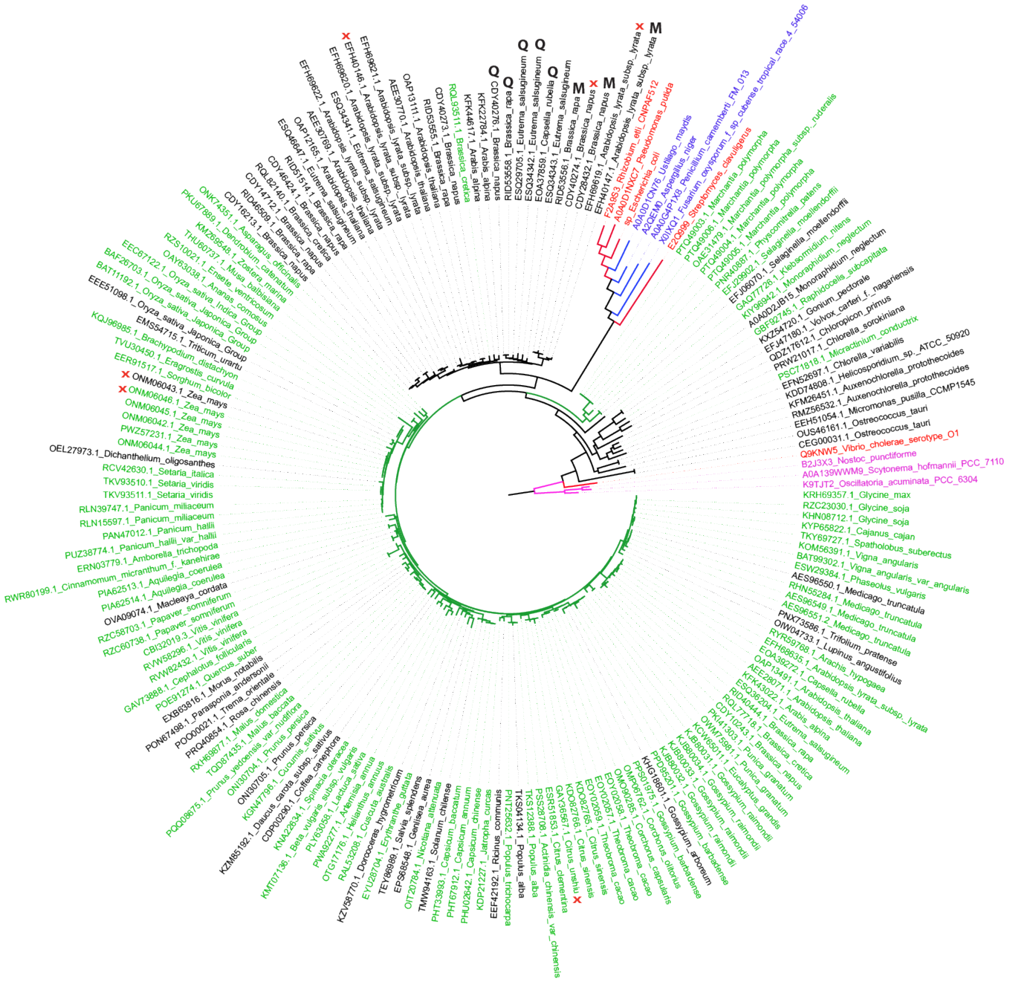

Supplementary Figure 9 - Phylogenetic relationship of plant DUF1338 homologs.

Phylogenetic tree showing plant DUF1338 containing proteins in addition to bacterial (red), cyanobacterial (pink), and fungal (blue) homologs. Green leaves show homologs that either have a predicted mitochondria or chloroplast localization sequence, while black leaves do not have any predicted localization tag. Red X's show proteins without a conserved "HHE" triad, while M and Q show proteins that have substitutions at the predicted specificity residue.

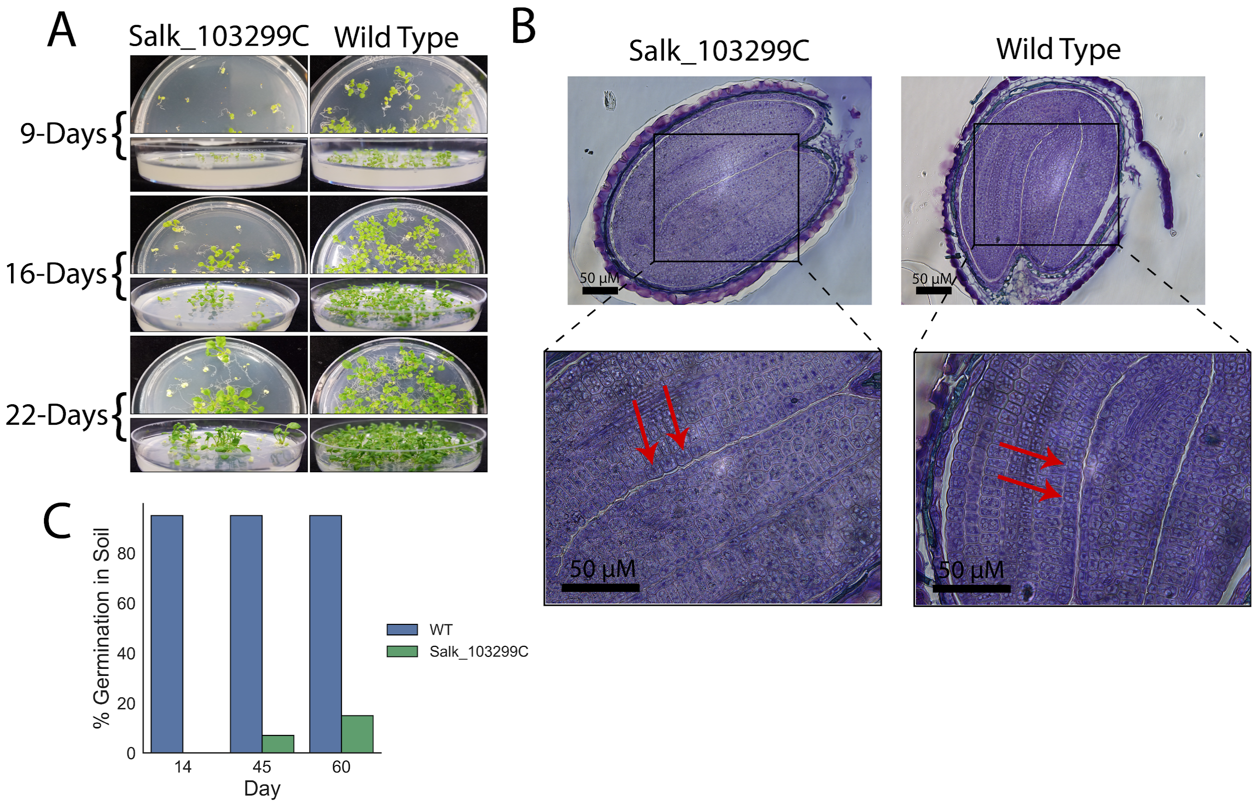

Supplementary Figure 10 - Germination rates of WT *A. thaliana* and Salk_103299C mutants. A) Arabidopsis seedlings germination and growth on germination medium at 9, 16, or 22 days after incubation. **B)** Toluidine blue stained histopathology of mature seeds from Salk_103299C mutant (left) and wild type seeds of *A. thaliana* (right). Sections are shown at 20x (top panels) and 40x (bottom panels) magnification. Oil bodies appear as non-toluidine O staining droplets within the cells of the plant embryo. Salk_103299C mutants appeared to have smaller and more numerous bodies compared to the wild type. Red arrows highlight cells where phenotype is visible. **C)** Germination rates of Salk_103299C relative to WT *A. thaliana*. Salk_103299C mutants showed delayed germination relative to WT *A. thaliana*. No mutants germinated 14 days after planting while 95% of WT seeds had germinated, n=96. 60 days after planting only 15% of mutant seeds had germinated.

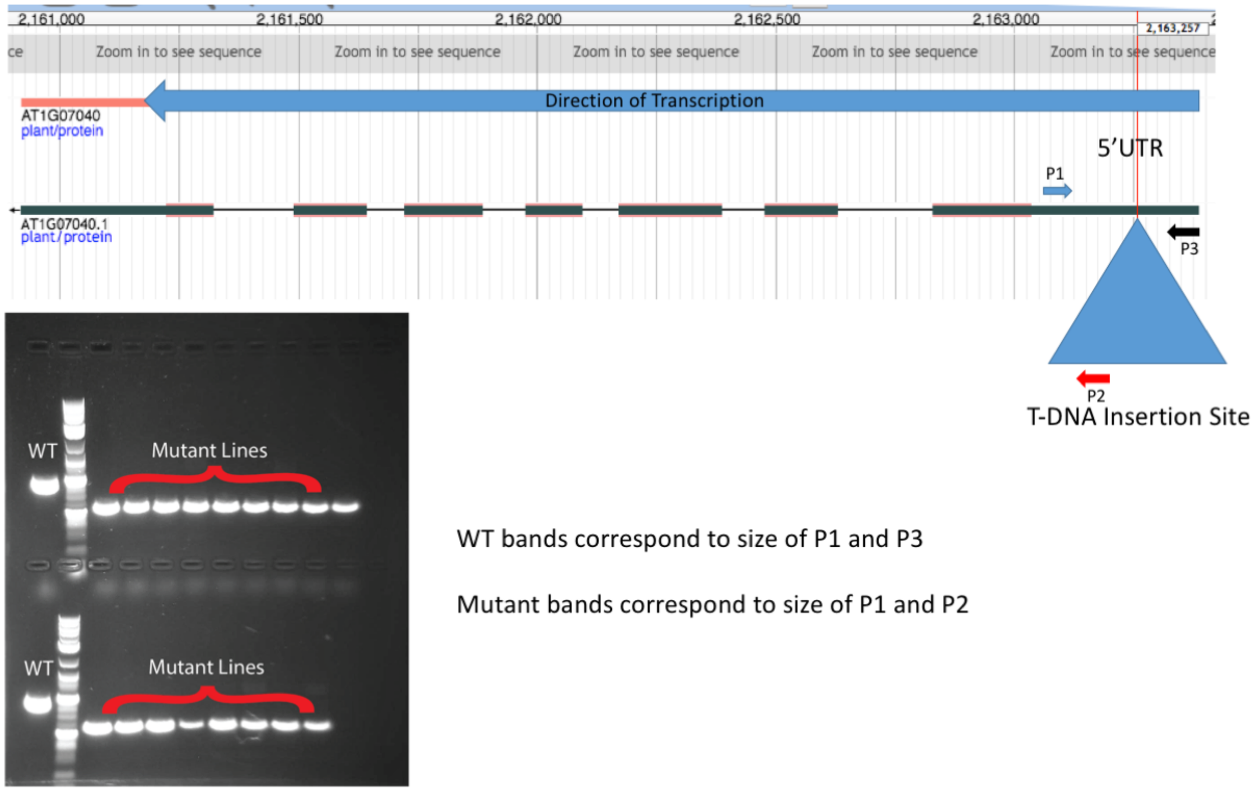

Supplementary Figure 11: Position of T-DNA insertion and confirmation of SALK lines - The above diagram shows the location of the T-DNA (represented by the blue triangle) insertion from SALK line Salk_103299C in the 5'UTR region upstream of the CDS. Individual plant genotypes were confirmed via 3 primer PCR. Primers P1 and P3 bind downstream and upstream of the T-DNA insertion relative to the orientation of the CDS, while primer P2 binds inside to T-DNA on the opposite strand as P1. Gel image shows bands amplified that correspond to the correct predicted amplicon of P1 and P3 to the left of the ladder, and to the right are bands that show one band at the size predicted to be amplified by P1 and P2. Each lane represents a single plant and the presence of one band confirms homozygosity.

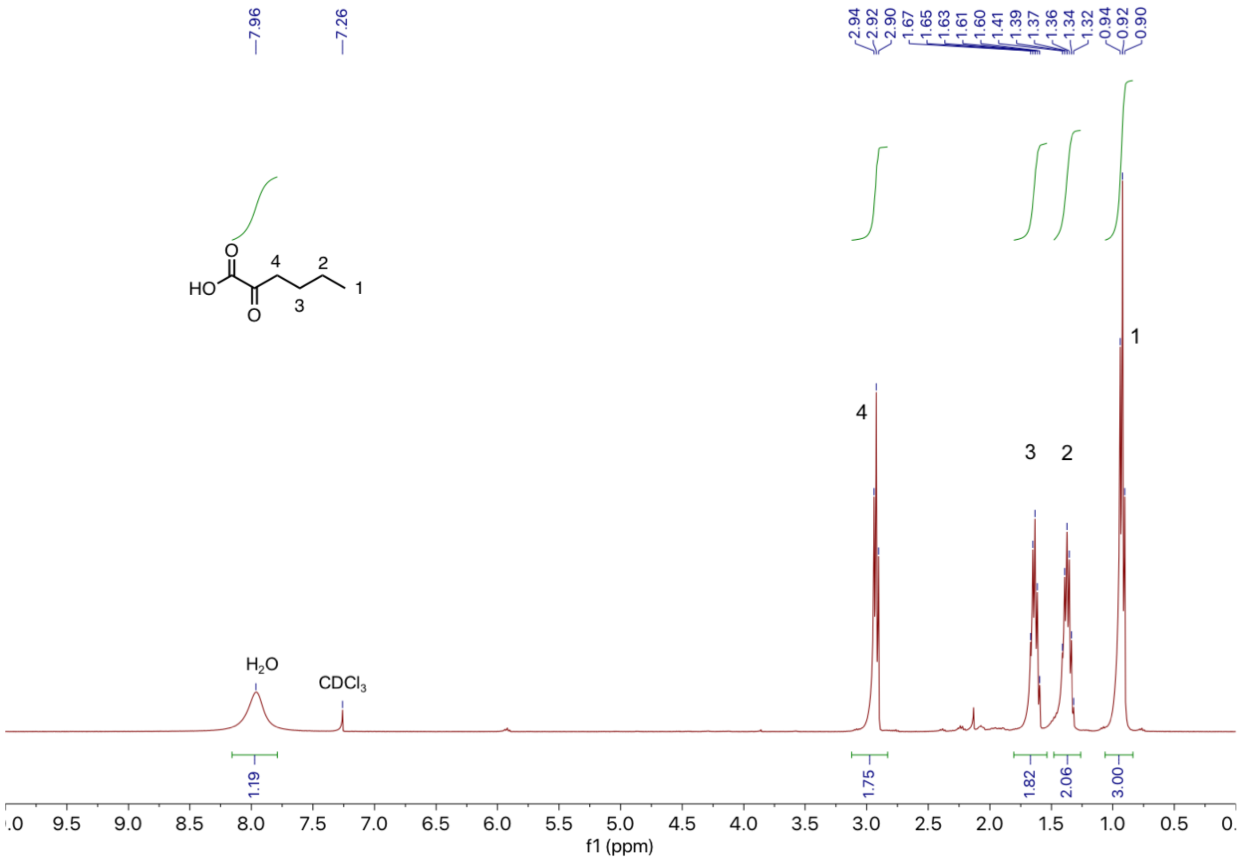

Supplementary Figure 12: ^{1}H NMR spectrum of 2-oxohexanoic acid. Signals corresponding to each set of ^{1}H nuclei are labeled. Detailed synthetic

Supplementary Tables

Supplementary Table 1- Distribution of DUF1338 proteins across domains of life and conservation of key residues. Table shows the conservation of the "HHE" metal binding triad, as well as the distal carboxylic acid coordinating R74 residue in homologs of DUF1338 family proteins across plants, bacteria, and fungi.

	DUF1338	HHE Triad	R74
Total	2417	2070	2058
Plant	150	122	114
Bacteria	2074	1773	1769
Fungi	184	172	172

Supplementary Table 2 - ATTED-II correlation of known lysine catabolic genes in *A. thaliana* with HglS homolog AT1G07040 and the 2KGD complex. Enzymes catalyzing reactions from Supplementary Figure 8 were analyzed for their coexpression with either HglS or 2KGD in *A. thaliana* and reported by their Mutual Rank Index.[3] HglS was highly correlated with all known lysine catabolic enzymes (column AT1G07040), while there was little correlation with the 2KGD complex (column AT5G65750). AT5G65750* represents the 2KGD complex E1 subunit only. The FLO7 homolog AT1G27030 however does not show co-expression with known lysine catabolic enzymes.

Reaction	Enzyme	AT Acc. No.	AT1G07040	AT5G65750	AT1G27030
1	Lysine-2KG reductase/saccharopine dehydrogenase	AT4G33150	3	1432.7	>2000
2	Aldehyde Dehydrogenase	AT1G54100	1.8	954.6	>2000
3	Gamma-aminobutyrate transaminase	AT3G22200	6.9	1955.9	1818.7
4	2-Oxoglutarate dehydrogenase complex	AT5G65750*	785.2	NA	>2000
5	Isovaleryl-coA dehydrogenase	AT3G45300	2.4	>2000	>2000
6	D-2-Hydroxyglutarate dehydrogenase	AT4G36400	15.5	>2000	>2000
7	D-2-Hydroxyglutarate synthase	AT1G07040	NA	785.2	>2000

Supplementary Table 3 - Predicted localization of plant DUF1338 proteins in sequenced plant genomes - BLAST results from all reference plant genomes in Genbank identified 202 HglS homologs. The table shows the conservation of the number of homologs that have a conserved HHE triad, the number than have the conserved R74, the number that have predicted localization to the chloroplast only, predicted localization to the mitochondria only, have either chloroplast or mitochondria localization prediction, or have no signal prediction.

	Total	HHE	R74	Chlor Localized Only	Mito Localized Only	Either Prediction	No Signal Prediction
Plants	202	167	159	61	8	117	85

Supplementary Table 4: Strains and plasmids used in this study

Strain	Reference
E. coli DH10B	Novagen
E. coli BL21(DE3)	Novagen
Plasmids	
pET21b	Novagen
pET21b-PP_5260	[1]
pET21b-PP_5260 R74A	This work
pET21b-PP_5260 V402P	This work
pET21b-*FLO7*	This work
pET21b-*FLO7*Δ1-76	This work
pET21b-AT1G07040Δ1-56	This work
pET21b-*ydcJ*_ECO	This work

Supplementary Table 5: Primers used in this study

Primer Name	Sequence	Description
j5_00005_(pET21b)_forward	GCTGTGGTTTGCTTCACCACCACCACCACCACT GAGAT	For Gibson Assembly of *E. coli* ydcJ purification vector
j5_00006_(pET21b)_reverse	CCGTGATGCTGTTCGCCATATGTATATCTCCTT CTTAAAGTTAAACA	For Gibson Assembly of *E. coli* ydcJ purification vector
j5_00007_(ydcJ)_forward	GAAGGAGATATACATATGGCGAACAGCATCAC GGCG	For Gibson Assembly of *E. coli* ydcJ purification vector
j5_00008_(ydcJ)_reverse	GGTGGTGGTGGTGAAGCAAACCACAGCGACGT TTACTGC	For Gibson Assembly of *E. coli* ydcJ purification vector
j5_00019_(pET21)_forward	CACACCAGGTCTCAGGCACCACCACCACCACC AC	For Golden Gate Assembly of *A. thaliana* FLO7 purification vector
j5_00002_(pET21)_reverse	CACACCAGGTCTCATATGTATATCTCCTTCTTA AAGTTAAACAAAA	For Golden Gate Assembly of *A. thaliana* FLO7 purification vector
j5_00021_(full_cDNA_Athaliana_FL O7)_reverse	CACACCAGGTCTCATGCCCGGTTCGTCGGGAA AGCT	For Golden Gate Assembly of *A. thaliana* FLO7 purification vector
j5_00022_(trunc_cDNA_Athaliana_F LO7)_forward	CACACCAGGTCTCACATATGTCATCAAACAAT GTTTCTCGCGG	For Golden Gate Assembly of *A. thaliana* FLO7 purification vector
j5_00007_(pET21)_forward	CACACCAGGTCTCACTCACCACCACCACCACC AC	For Golden Gate Assembly of *O. sativa* FLO7 purification vector
j5_00003_(pET21)_reverse	CACACCAGGTCTCATATGTATATCTCCTTCTTA AAGTTAAACAAAA	For Golden Gate Assembly of *O. sativa* FLO7 purification vector
j5_00010_(Osativa_flo7_dna_trunc)_f orward	CACACCAGGTCTCACATATGGCGGCCGCTGCC TCT	For Golden Gate Assembly of *O. sativa* FLO7 purification vector
j5_00011_(Osativa_flo7_dna_trunc)_r everse	CACACCAGGTCTCATGAGCCGAGCGACGCGTC AACT	For Golden Gate Assembly of *O. sativa* FLO7 purification vector
ColE1-bb-F	CAGGTCTCA gacctacaccgaactgagatacctacagc	For Golden Gate assembly of HglS mutants
ColE1-bb-F	CAGGTCTCA ggtcgttcgctccaagctgg	For Golden Gate assembly of HglS mutants
5260-R74A-F	CAGGTCTCA catc GCC gtcggcaccgccgaagaact	For Golden Gate assembly of HglS mutant R74A
5260-R74A-R	CAGGTCTCA gatg gcgccgtgacgttcctg	For Golden Gate assembly of HglS mutant R74A
5260_V402P-F	CAGGTCTCA GCCGCCGAGTGCG	For Golden Gate assembly of HglS mutant V402P
5260_V402P-R	CAGGTCTCA cggcaggaagtcctcgta	For Golden Gate assembly of HglS mutant V402P

Supplementary Table 6: Summary of X-ray data collection statistics HglS apo-enzyme, HgIS substrate-bound and Flo7 substrate-bound.

	HglS *apo*	HgIS substrate-bound	Flo7 substrate-bound
Wavelength	1.00000	0.99999	0.97946
Resolution range	50.37 - 1.14 (1.181 - 1.14)	72.05 - 1.419 (1.47 - 1.419)	38.65 - 1.85 (1.916 - 1.85)
Space group	P 21 21 21	P 21 21 21	P 21 21 21
Unit cell	42.44 100.74 104.76 90 90 90	42.44 100.28 103.57 90 90 90	92.30 98.20 203.80 90 90 90
Total reflections	1121754 (103068)	593341 (56327)	1141314 (116637)
Unique reflections	164034 (16167)	84333 (8301)	156493 (15364)
Multiplicity	6.8 (6.4)	7.0 (6.8)	7.3 (7.6)
Completeness (%)	99.94 (99.40)	99.84 (99.10)	98.94 (98.68)
Mean I/sigma(I)	12.33 (2.10)	16.37 (1.36)	11.52 (1.39)
Wilson B-factor	13	15.71	29.19
R-merge	0.0615 (0.6688)	0.06148 (1.42)	0.1162 (1.592)
R-meas	0.06648 (0.7282)	0.06641 (1.538)	0.1251 (1.707)
R-pim	0.0249 (0.2833)	0.02487 (0.5859)	0.04555 (0.6084)
CC1/2	0.999 (0.666)	0.999 (0.736)	0.998 (0.517)
CC*	1 (0.894)	1 (0.921)	1 (0.825)
Reflections used in refinement	164033 (16166)	84229 (8250)	156474 (15364)
Reflections used for R-free	8263 (802)	4041 (367)	7752 (777)
R-work	0.1423 (0.2399)	0.1572 (0.2416)	0.1667 (0.3159)
R-free	0.1661 (0.2710)	0.1937 (0.3251)	0.1943 (0.3405)
CC(work)	0.965 (0.863)	0.966 (0.871)	0.963 (0.787)
CC(free)	0.962 (0.836)	0.948 (0.763)	0.957 (0.772)
Number of non-hydrogen atoms	4121	4265	11051
Macromolecules	3466	3569	9800
Ligands	1	2	24

Solvent	654	664	1123
Protein residues	434	450	1228
RMS(bonds)	0.012	0.008	0.011
RMS(angles)	1.23	0.870	1.086
Ramachandran favored (%)	98.83	97.76	98.03
Ramachandran allowed (%)	1.17	2.26	1.89
Ramachandran outliers (%)	0	0.00	0.08
Rotamer outliers (%)	0.55	1.33	0
Clashscore	1.74	2.11	2.21
Average B-factor	22.97	23.90	37.70
Macromolecules	21.15	22.25	36.15
Ligands	13.70	19.49	89.45
Solvent	32.63	34.77	45.40

References

1. Thompson, M. G. *et al.* Massively Parallel Fitness Profiling Reveals Multiple Novel Enzymes in Pseudomonas putida Lysine Metabolism. *MBio* 10, (2019).
2. Hildebrandt, T. M., Nunes Nesi, A., Araújo, W. L. & Braun, H.-P. Amino acid catabolism in plants. *Mol. Plant* 8, 1563–1579 (2015).
3. Obayashi, T., Aoki, Y., Tadaka, S., Kagaya, Y. & Kinoshita, K. ATTED-II in 2018: A Plant Coexpression Database Based on Investigation of the Statistical Property of the Mutual Rank Index. *Plant Cell Physiol.* 59, e3 (2018).
