# Supplementary material for: An iron (II) dependent oxygenase performs the last missing step of plant lysine catabolism
Source: Nat Commun. 2020 Jun 10;11:2931. doi: 10.1038/s41467-020-16815-3 (PMC7286885; doi:10.1038/s41467-020-16815-3)
Supplement: Supplementary file 2 — Description of Additional Supplementary Files [file 41467_2020_16815_MOESM2_ESM.pdf]

**Description of Additional Supplementary Files**

**File name:** Supplementary Dataset 1

**Description:** VAST search results of HglS
